# Supplementary material for: B7-H3 Associates with IMPDH2 and Regulates Cancer Cell Survival
Source: Cancers (Basel). 2023 Jul 7;15(13):3530. doi: 10.3390/cancers15133530 (PMC10340635; doi:10.3390/cancers15133530)
Supplement: Supplementary file 1 [file cancers-15-03530-s001.zip › cancers-2473846-supplementary.pdf]

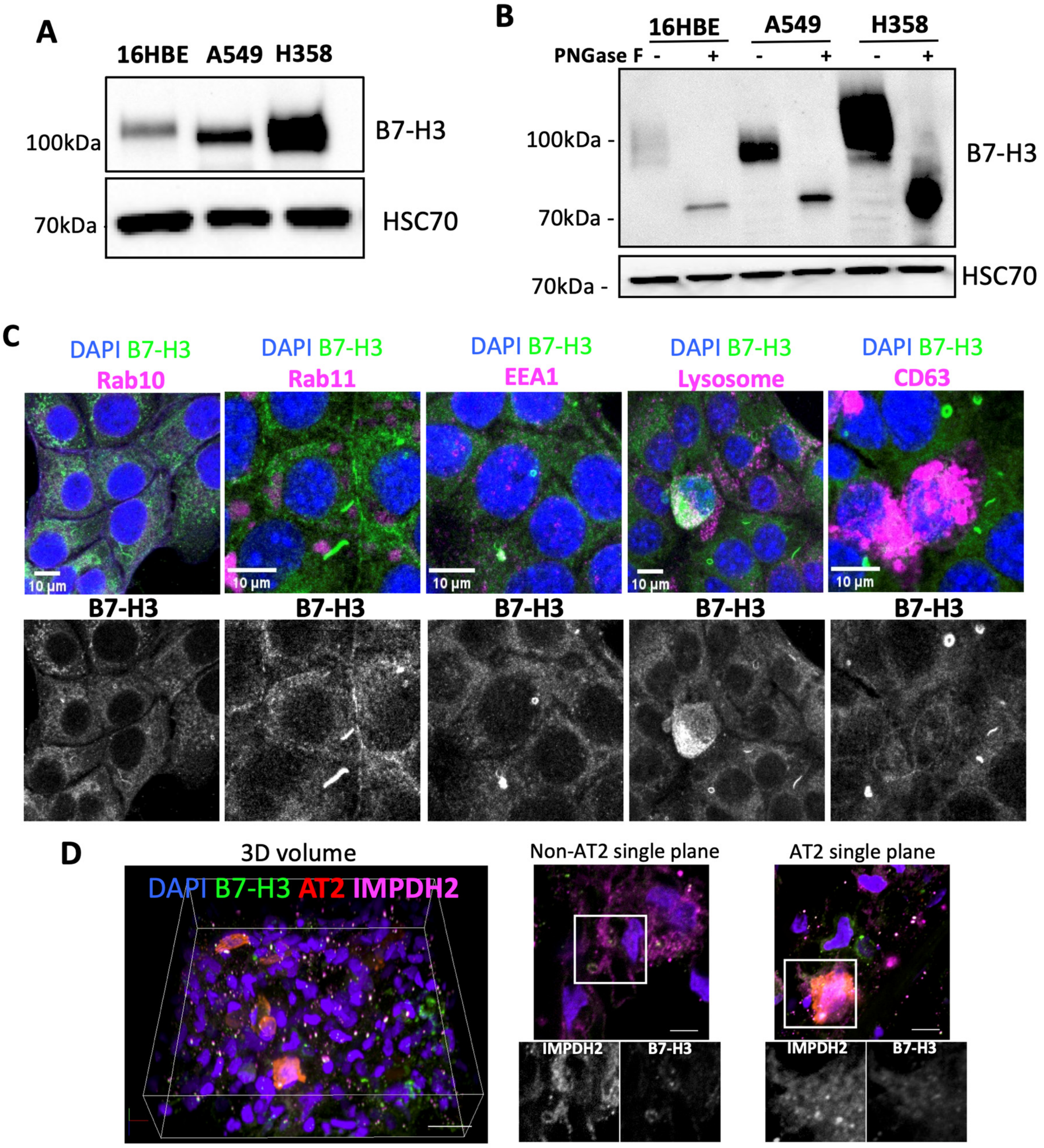

Figure S1: B7-H3 localises to RR and expression is increased in lung cancer cell lines compared to normal lung epithelium

A) Western blot of lysates from 16HBE, A549 and H348 cells probed for B7-H3 and HSC70 **(B)** Western blot of lysates from specified control cells (-) or those cells treated with PNGaseF glycosylation inhibitor (+) probed for B7-H3 and HSC70. Representative of 4 experiments. **(C)** Representative confocal images of 16HBE cells stained for nuclei (blue), B7-H3 (green middle panel) and either Rab10, Rab11 or EEA1 (magenta and far right panel). Lysotracker or overexpressed with CD63-GFP (magenta and far right panel). Scale bars 10µm. **(D)** representative 3D volume of human slice slice stained for specified markers. Scale bar 50µm. Images on right show x,y single slices images of example RR containing IMPDH2 and B7-H3 in non-AT2 epithelial cells (left) and AT2 cells (right). Scale bars 10µm

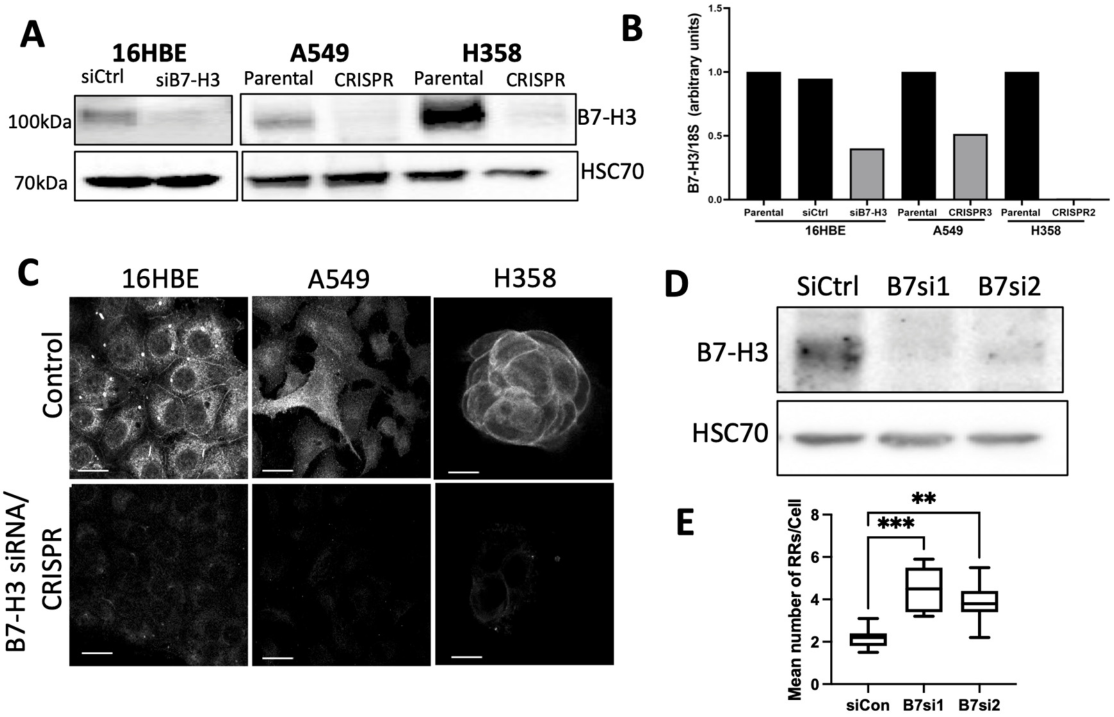

Figure S2: Depletion of B7-H3 in cell lines

(A) Western blot of lysates from B7-H3 knockdown cells (siRNA in 16HBE, CRISPR in A549 and H358 cells). **(B)** Quantification of B7-H3 mRNA levels in knockdown cells by qPCR. **(C)** Representative images of control and B7-H3 knockdown cells stained for endogenous B7-H3. Scale bars 10µm. **(D)** Western blot showing efficiency of additional single siRNAs targeting B7-H3. **(E)** Quantification of RR formation in B7-H3 knockdown 16HBE cells (as in D) from images of cells fixed and stained for IMPDH2. ** $p<0.01$, *** $p<0.005$

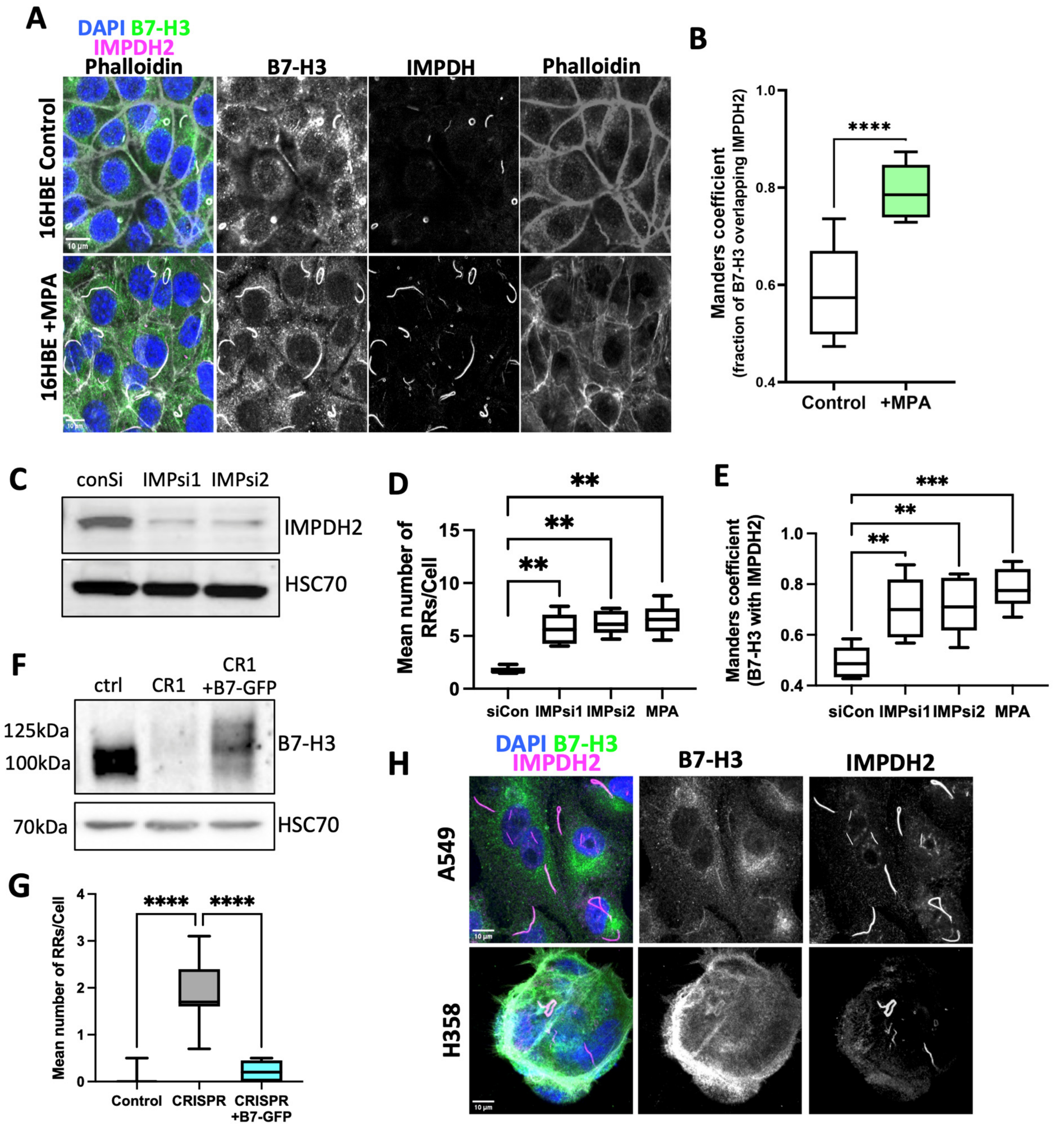

Figure S3: IMPDH2 inhibition increases RR assembly and B7-H3:IMPDH2 colocalisation

(A) Representative confocal images of 16HBE cells stained for B7-H3 (green) and IMPDH2 (magenta) post 48 h of MPA treatment. **(B)** Quantification of co-localisation of B7-H3 and IMPDH2 at RR from cells stained for both proteins and treated with MPA for 48 h. **(C)** Representative blot of 16HBE cells treated with control siRNA or two individual siRNAs targeting IMPDH2 (IMPsi1 or 2). **(D)** Quantification of RR/cell from cells as in (C); MPA included as positive control. **(E)** Quantification of co-localisation of B7-H3 and IMPDH2 at RR from cells stained for both proteins and treated with specified siRNA or MPA for 48h. **(F)** Western blot of A549 parental (ctrl), CRISPR or CRISPR+B7-H3-GFP probed for B7-H3. **(G)** Quantification of RR/cell from cells as in (F). **(H)** Representative confocal images of A549 and H358 cells treated with MPA for 48 h and stained for nuclei (blue), B7-H3 (green middle panel) and IMPDH2 (magenta and far right panel). Scale bars 10µm. Graphs shown as mean +/- SEM. Significance assessed by t-test or one-way ANOVA; **p<0.01, *** p<0.005, ****p<0.0001 . Scale bars 10µm

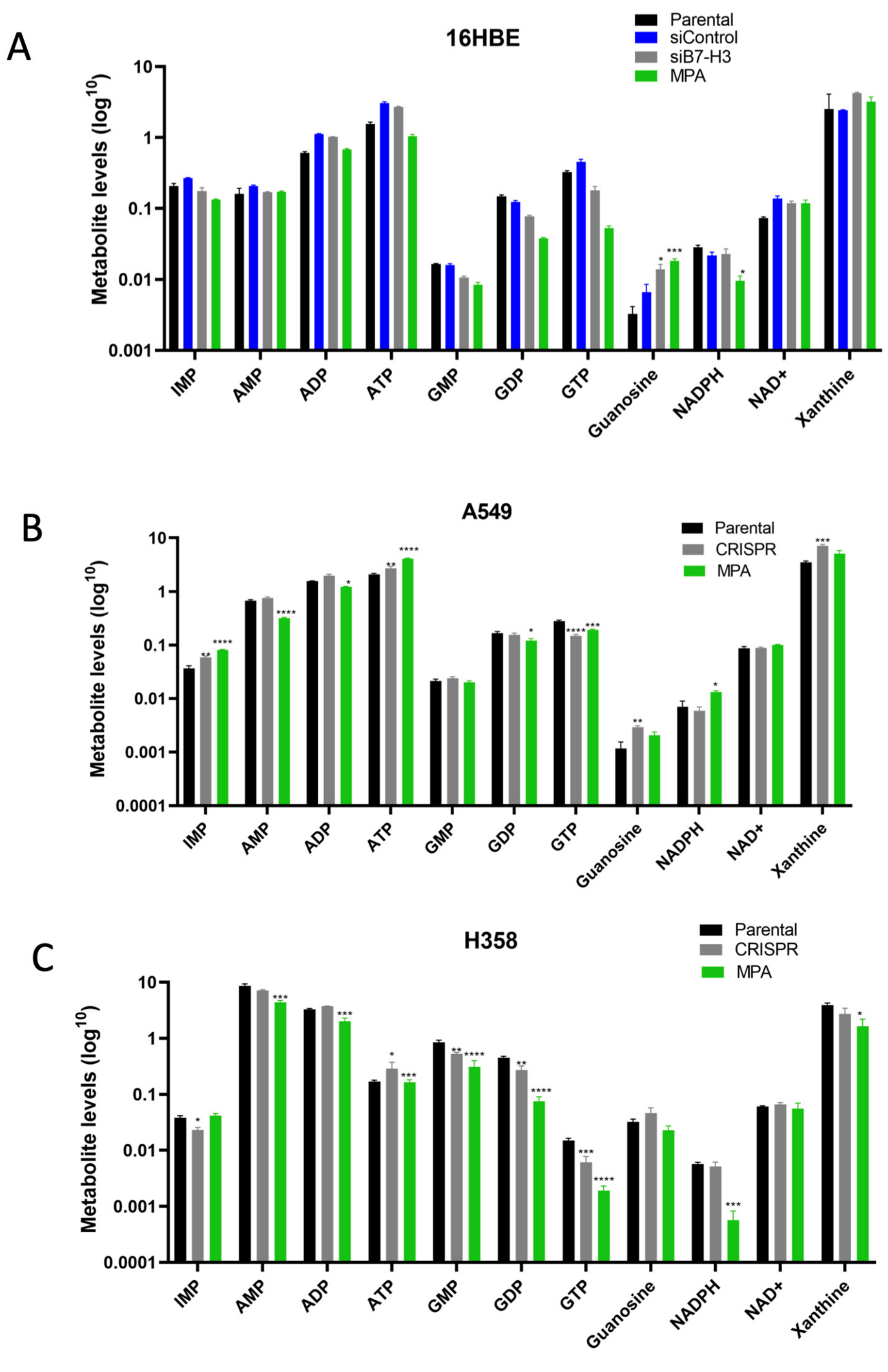

Figure S4: Reducing B7-H3 leads to reduced IMPDH2 pathway metabolites

Graph of key changes in metabolites levels of the IMPDH2 pathway analysed by mass spectrometry in parental/siControl, B7-H3 siRNA/CRISPR knockdowns and MPA treated in (A) 16HBE, (B) A549 and (C) H358 cells. Y-axis values represent a ratio of area of each peak of metabolite to the peak area of internal standard that with the closest retention time and/or structure. Note these are provided as a log^{10} scale due to the broad range of concentrations of each metabolite found in cells.

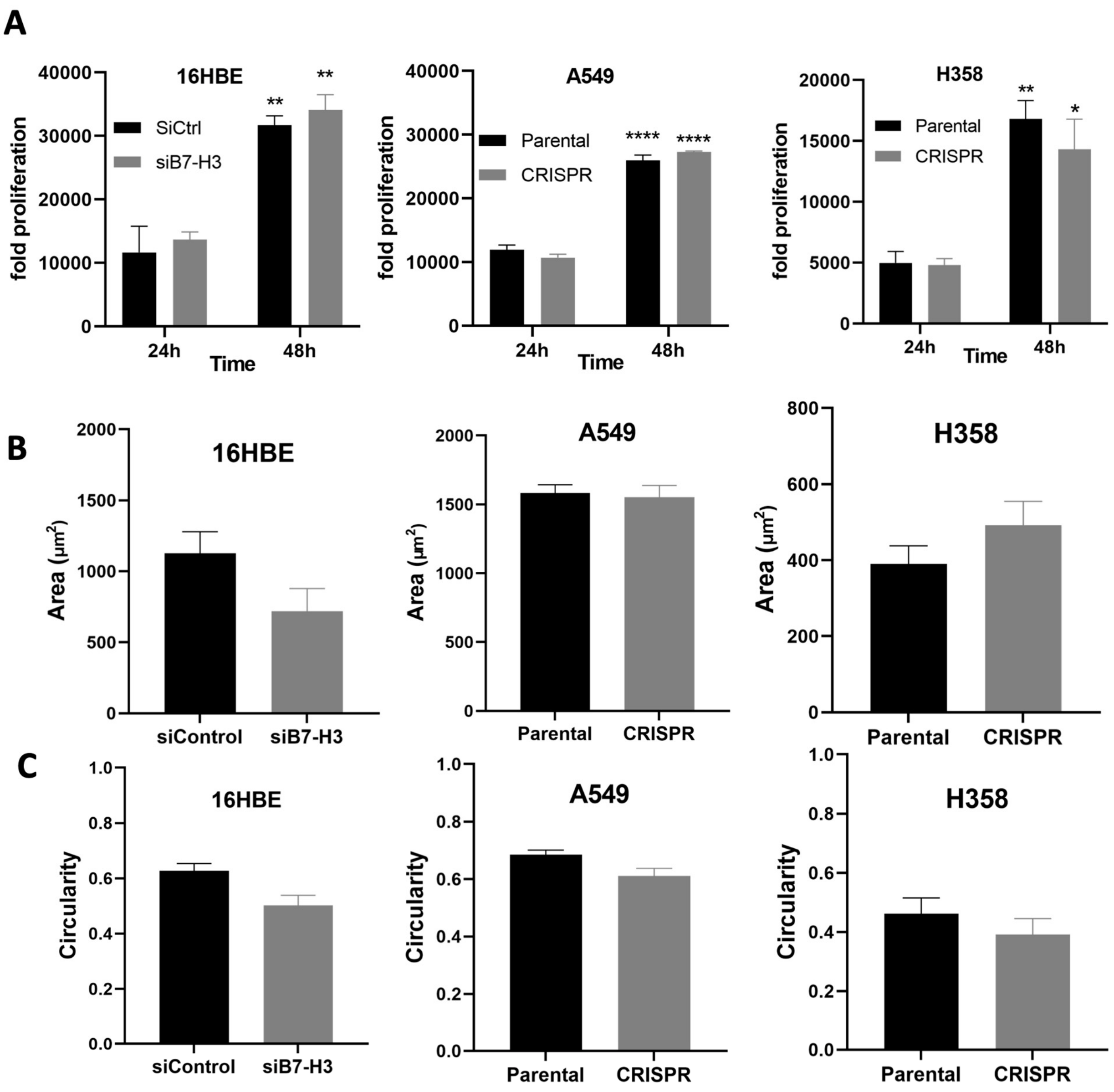

Figure S5: B7-H3 knockdown does not affect proliferation or shape in 2D.
A) Equal numbers of parental/siControl and B7-H3 siRNA/CRISPR cells were plated and fixed at 24h and 48h. Nuclei were stained with DAPI and fold proliferation was determined. **(B)** Quantification of cell area and **(C)** shape represents 20 cells/cell line per experiment pooled from 3 independent experiments. Values are plotted as mean +/- SEM. One-way ANOVA or t-test was used to determine statistical significance. *=p<0.05. **=p<0.01 ****=p<0.0001.

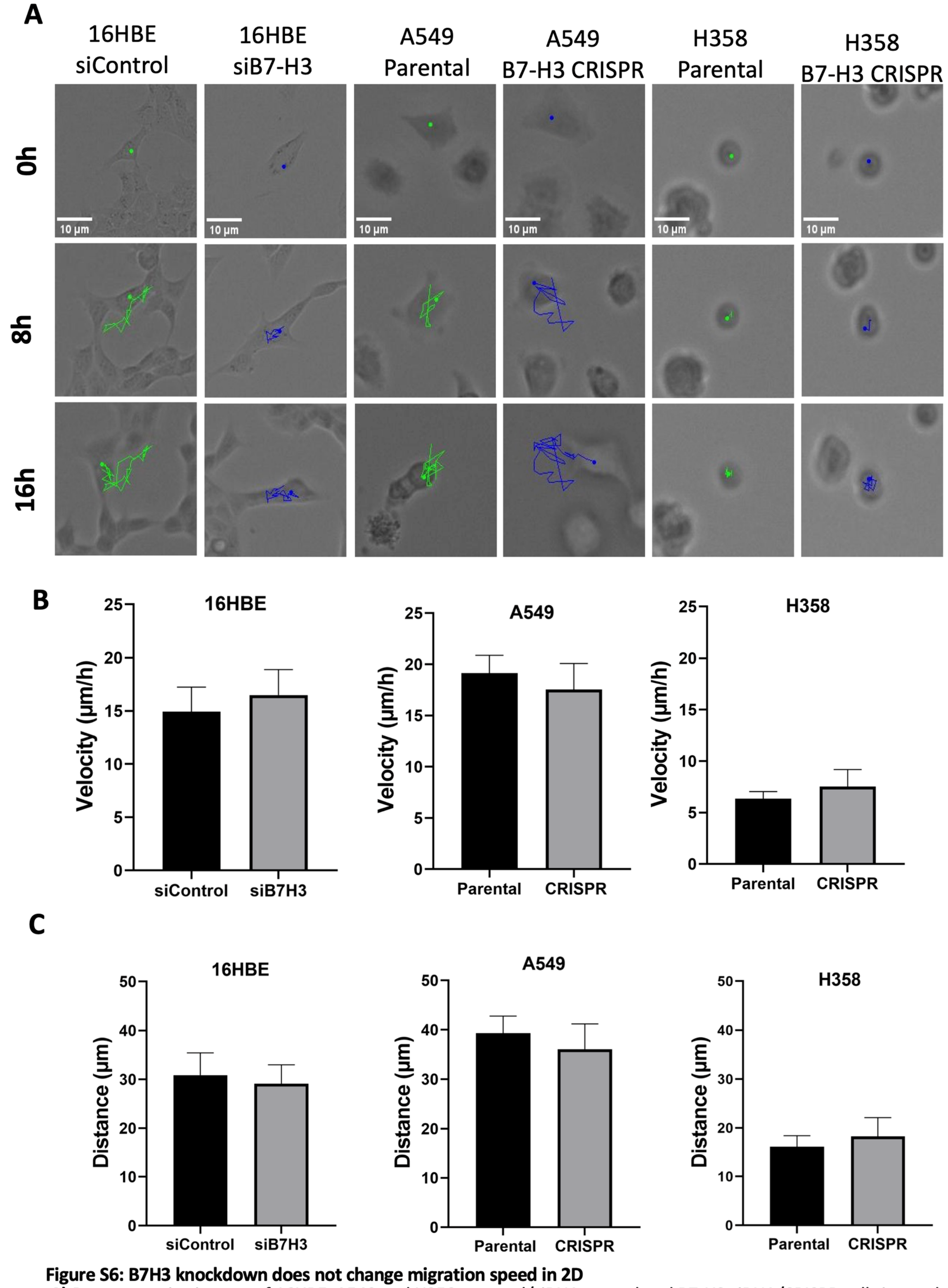

Figure S6: B7H3 knockdown does not change migration speed in 2D
A) Representative images of 16HBE, A549 and H358 parental/siRNA control and B7-H3 siRNA/CRISPR cells imaged over 16h. **(B,C)** Quantification of the velocity **(B)** and migrated distance **(C)** from movies as in A. At least 100 cells tracked per condition. One experiment shown, representative of 3 independent experiments. Graphs shown as mean +/- SEM. Significance assessed by t-test.

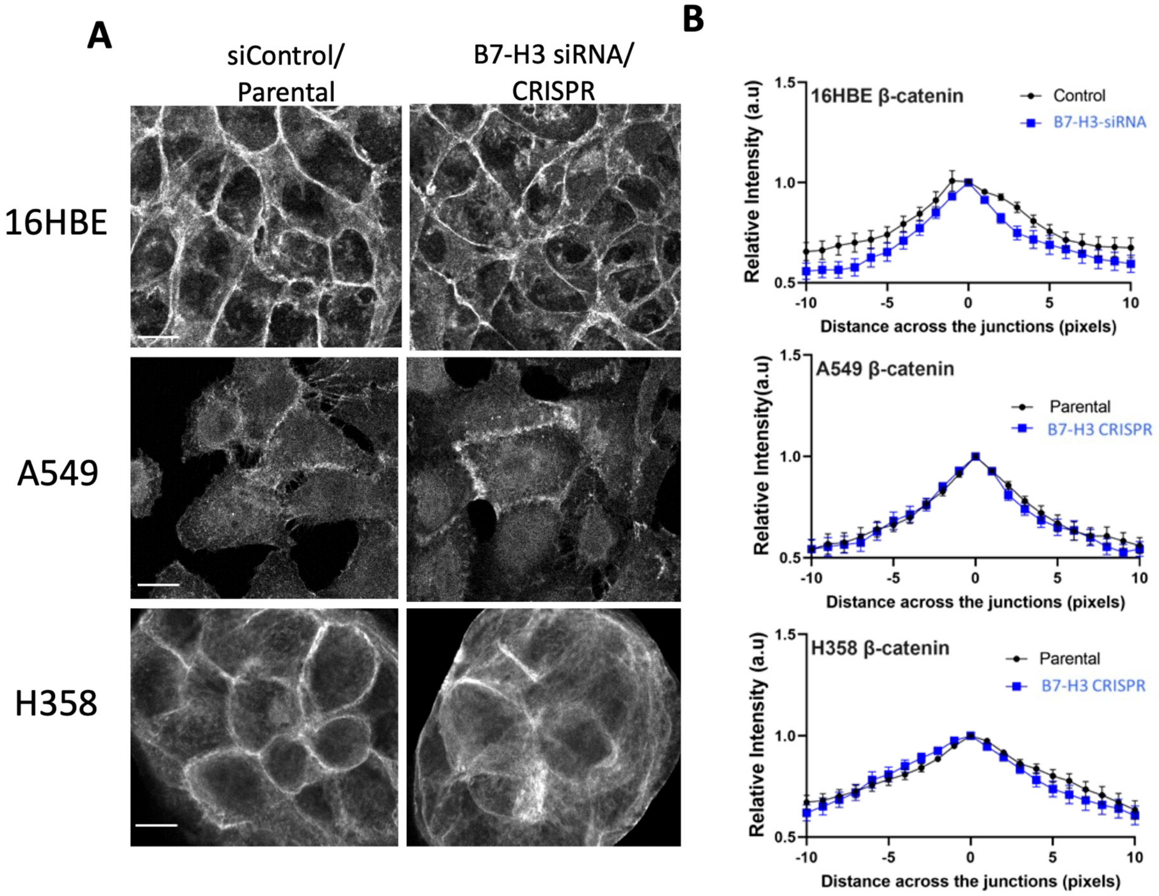

Figure S7: B7-H3 knockdown does not alter levels or localisation of β-Catenin
(A) Representative confocal images of siRNA control or Parental lines compared to B7-H3 siRNA/CRISPR cells fixed and stained for β-Catenin. **(B)** Quantitative line scan analysis of β-catenin signal intensity at junction. Data pooled from 20 junctions per cell line from 5 different fields of view of 3 independent experiments. Data shown is representative of one independent experiment showing mean +/- SEM. One-way ANOVA was used to compare statistical significance. Scale bars 10μm.

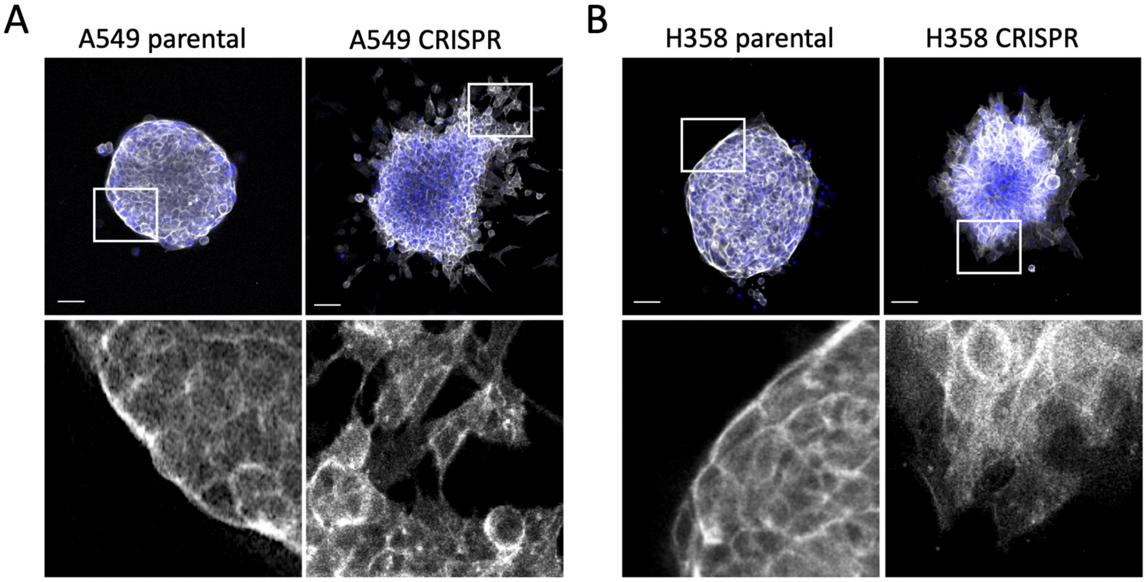

Figure S8: B7-H3 depletion leads to reduced F-actin intensity at the boundary of 3D spheroids
Representative confocal images of A549 **(A)** and H358 (**B**) Parental and B7-H3 CRISPR cells fixed and stained for DAPI 9blue) and phalloidin (white). Inset panels from boxed regions shown in main panel images are shown below for F-actin only, Scale bars 10µm.

Figure 3A

B7-H3 IMPDH2

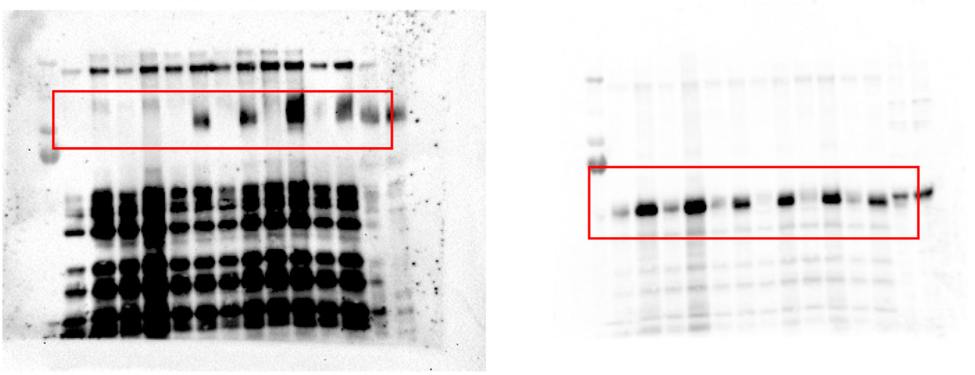

Figure 3C

IMPDH2 B7-H3

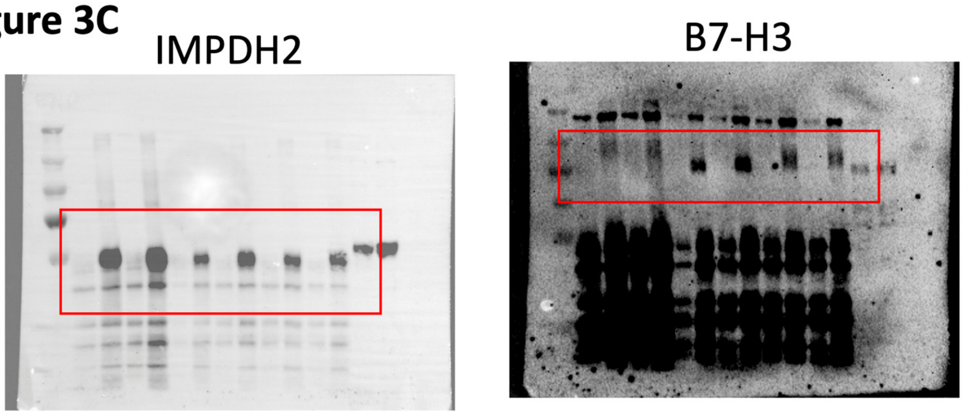

Figure 8A

ZO-1

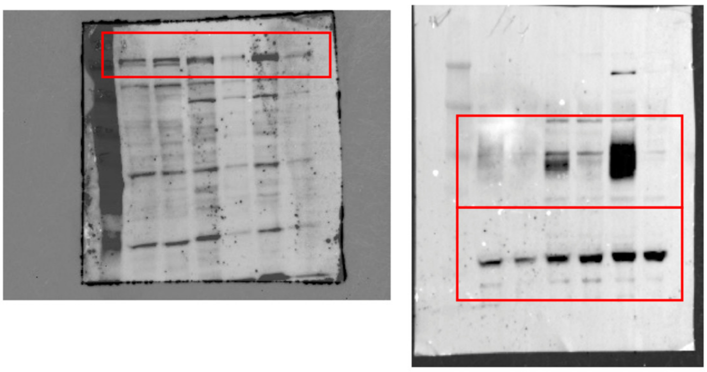

B7-H3

HSC70

Figure S9: Whole western blots for Main Figures

Figure S1A

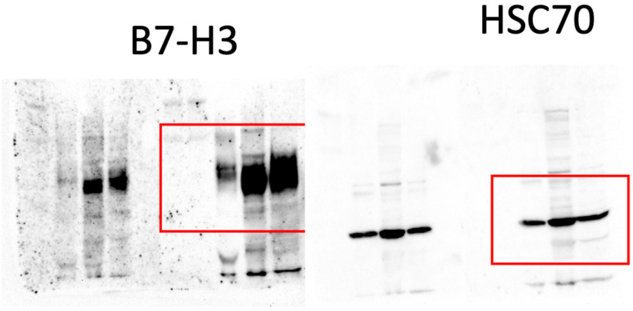

Figure S1B

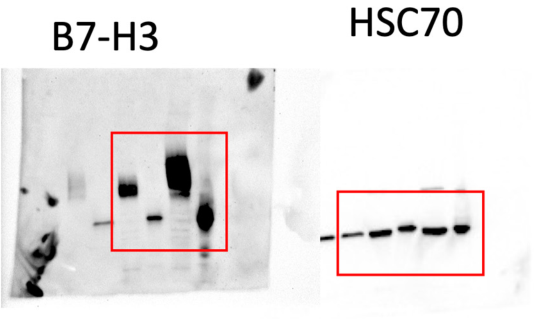

Figure S2A

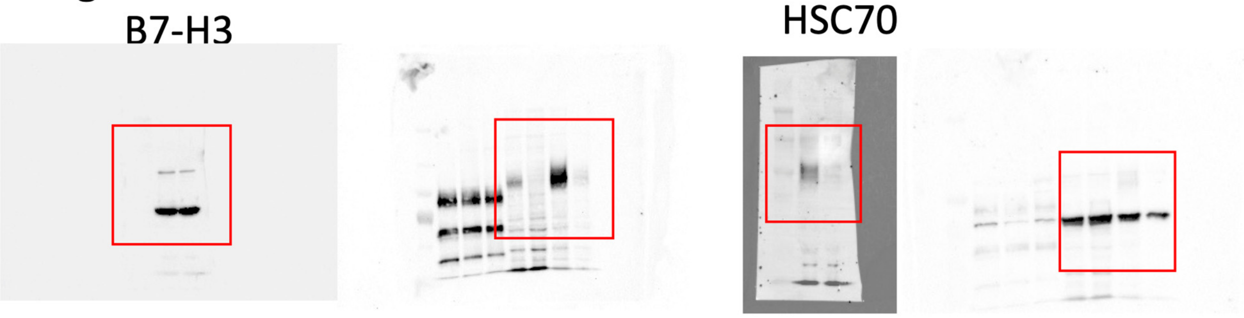

Figure S3C

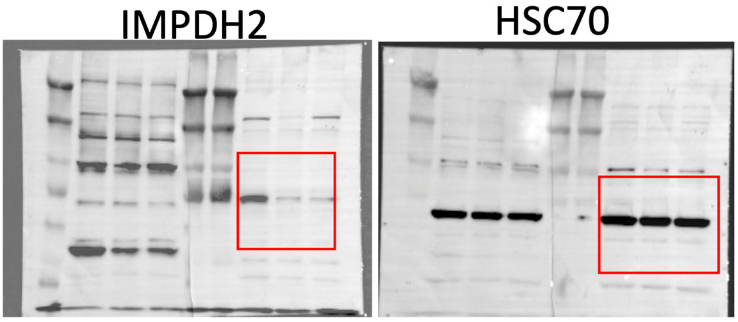

Figure S3F

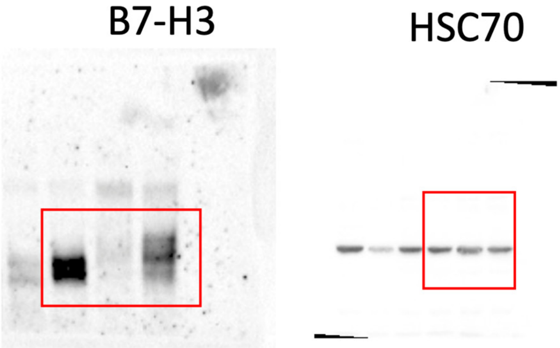

Figure S10: Whole western blots for Supplementary Figures
